# Crystal structure of 4-meth­oxy-*N*-(piperidine-1-carbono­thio­yl)benzamide

**DOI:** 10.1107/S2056989017013317

**Published:** 2017-09-25

**Authors:** Khairi Suhud, Siti Aishah Hasbullah, Musa Ahmad, Lee Yook Heng, Mohammad B. Kassim

**Affiliations:** a School of Chemical Sciences and Food Technology, Faculty of Science and Technology, Universiti Kebangsaan Malaysia, 43600 Selangor, Malaysia; b Department of Chemistry, Mathematic & Natural Science Faculty, Universitas Syiah Kuala, Banda Aceh, 23111, Indonesia; c Chemical Technology Program, Faculty of Science Technology, Universiti Sains Islam Malaysia, Bandar Baru Nilai, 71800 Nilai, Negeri Sembilan; d Fuel Cell Institute, Universiti Kebangsaan Malaysia, 43600 Selangor, Malaysia

**Keywords:** crystal structure, benzoyl­thio­urea, piperidine, pyrrolidine, benzamide, anti-cancer, hydrogen bonding, C—H⋯π inter­actions, offset π–π inter­actions

## Abstract

In the title compound, C_14_H_18_N_2_O_2_S, the piperidine ring has a chair conformation. Its mean plane is twisted with respect to the 4-meth­oxy­benzoyl ring, with a dihedral angle of 63.0 (3)°. The central N—C(=S)—N(H)—C(=O) bridge is twisted with an N—C—N—C torsion angle of 74.8 (6)°. In the crystal, mol­ecules are linked by N—H⋯O and C—H⋯O hydrogen bonds, forming chains along the *c*-axis direction. Adjacent chains are linked by C—H⋯π inter­actions, forming layers parallel to the *ac* plane. The layers are linked by offset π–π inter­actions [inter­centroid distance = 3.927 (3) Å], forming a supra­molecular three-dimensional structure.

## Abstract 2

In the title benzoyl­thio­urea derivative (MPiCB), the piperidine ring has a chair conformation and its mean plane is inclined to the 4-meth­oxy­benzene ring by 63.0 (3)°.

## Chemical context   

Benzoyl­thio­urea compounds exhibit anti-inflammatory (Brachmachari & Das, 2012 ▸), anti-cancer, anti-diabetic and anti-virus activity (Kovačková *et al.*, 2011 ▸), and have applications as ionic sensors (Suhud *et al.* 2015*b* ▸) and pharmaceutical drugs (Watson *et al.*, 2000 ▸). Benzoyl­thio­urea mol­ecules containing thio­amide (NH—C=S) and carbonyl (C=O) electron-rich donating groups facilitate the formation of coordination bonds with metal ions such as Co^3+^ (Tan *et al.*, 2014 ▸), Ru^2+^ (Małecki & Nycz, 2013 ▸), Ag^+^ (Isab *et al.*, 2010 ▸) and Ni^2+^ (Arslan *et al.*, 2006 ▸). Bivalent and trivalent metal ions prefer to coordinate *via* the S and O atoms from the thiono and carbonyl units, respectively, but monovalent metal ions tend to coordinate *via* the S atom.
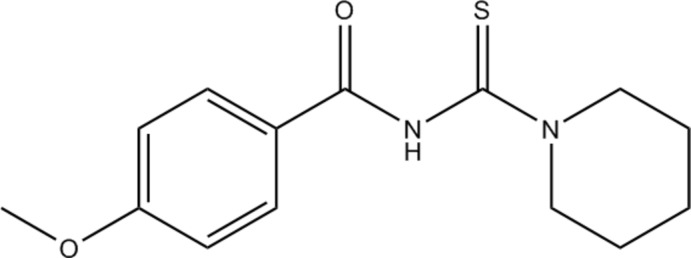


Herein, we report on the crystal structure of 4-meth­oxy-*N*-(piperidine-1-carbono­thio­yl)benzamide (MPiCB) and its chemical structural data in comparison with the previously reported compound 4-meth­oxy-*N*-[(pyrrolidin-1-yl)carbono­thio­yl]benzamide (MPCB; Suhud *et al.*, 2015*a* ▸,*b* ▸).

## Structural commentary   

**Figure 1:**
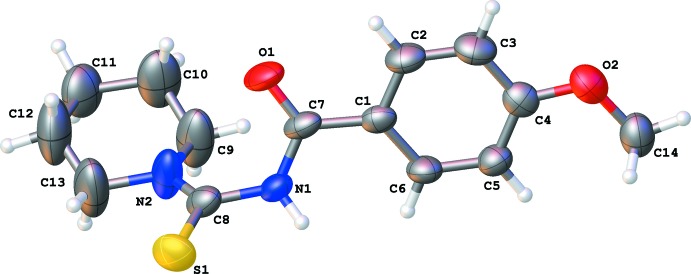
A view of the mol­ecular structure of the title compound (MPiCB), with the atom labelling. Displacement ellipsoids are drawn at the 50% probability level.

The mol­ecular structure of the title compound, MPiCB, is illustrated in Fig. 1 ▸. The geometrical parameters are similar to those observed for 4-meth­oxy-*N*-[(pyrrolidin-1-yl)carbo­thio­yl]benzamide (MPCB; Suhud *et al.* 2015*a* ▸). The 4-meth­oxy­benzoyl and piperidine fragments adopt a *trans*–*cis* conformation with respect to the thiono S atom across the C8—N1 bond, with the piperidine ring having a chair conformation. The mean plane of the piperidine ring is twisted with respect to the 4-meth­oxy benzoyl ring with a dihedral angle of 63.0 (3)°. The central N—C(=S)—N(H)—C(=O) bridge is twisted with an N2—C8—N1—C7 torsion angle of 74.8 (6)°. The meth­oxy group lies in the plane of the benzene ring, with the C14—O2—C4—C3 torsion angle being 180.0 (4)°.

## Supra­molecular features   

**Figure 2:**
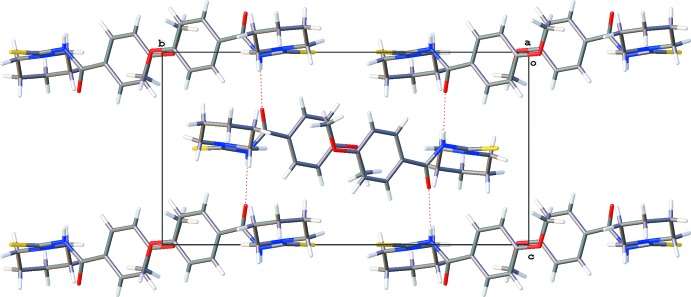
A view along the *a* axis of the crystal packing of the title compound (MPiCB). Hydrogen bonds (see Table 1 ▸) are shown as dashed lines.

**Figure 3:**
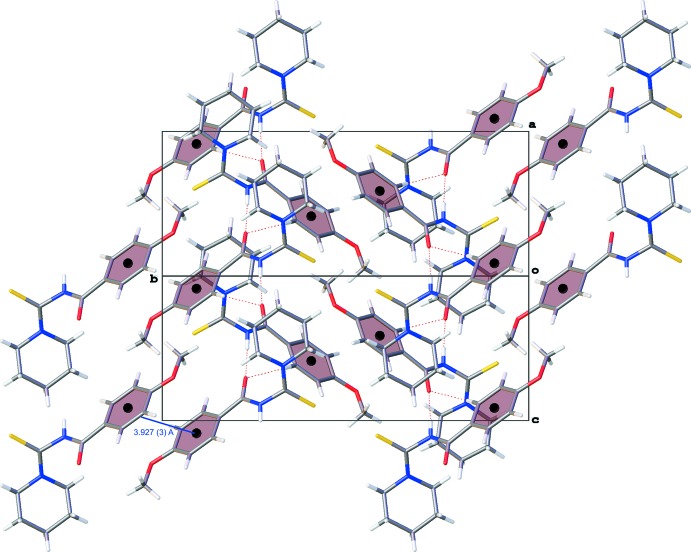
A view normal to the *ac* plane of the crystal packing of the title compound (MPiCB), showing the offset π–π stacking inter­actions that add further stabilization to the crystal structure, and the hydrogen bonds (dashed lines).

In the crystal of MPiCB, neighbouring mol­ecules are linked by N—H⋯O and C—H⋯O hydrogen bonds, forming chains along the *c*-axis direction (Table 1 ▸ and Fig. 2 ▸). Adjacent chains are linked by C—H⋯π inter­actions, involving a piperidine H atom and the π electrons of the benzene ring, forming layers parallel to the *ac* plane (Table 1 ▸ and Fig. 3 ▸). The layers are linked by offset π–π stacking inter­actions involving the benzene rings, forming a supra­molecular three-dimensional structure as illustrated in Fig. 3 ▸ [*Cg*⋯*Cg* ^i^ = 3.927 (3) Å; *Cg* is the centroid of the C1–C6 ring; inter­planar distance = 3.517 (2) Å; slippage = 1.747 Å; symmetry code: (i) −*x*, −*y* + 2, −*z* + 2].

## Database survey   

A search of the Cambridge Structural Database (Version 5.38, update May 2017; Groom *et al.*, 2016 ▸) for the 4-meth­oxy-*N*-(carbono­thio­yl)benzamide skeleton gave 37 hits. Two compounds are of particular inter­est, namely 4-meth­oxy-*N*-(pyrrolidin-1-ylcarbono­thio­yl)benzamide (DUDYOS; Suhud *et al.*, 2015*a* ▸) mentioned previously (MPCB), and *N*-(2,6-di­meth­yl­piperidine-1-carbono­thio­yl)-3,4,5-tri­meth­oxy­benz­amide (HESLEX; Dillen *et al.*, 2006 ▸). The 4-meth­oxy­benzoyl ring and the mean plane of the piperidine ring in MPiCB form a smaller angle [63.13 (3)°] compared with the angle of 72.60 (14)° for similar mean planes found in DUDYOS (Suhud *et al.* 2015*a* ▸). The bond lengths for C8=S1 [1.651 (4) Å] and C7=O1 [1.226 (4) Å] in MPiCB are comparable to those observed for DUDYOS [C=S = 1.662 (2) Å and C=O = 1.220 (2) Å]. Other bond lengths and angles in the MPiCB mol­ecule are comparable with those reported for DUDYOS and *N*-(pyrrolidin-1-ylcarbo­thio­yl)benzamide (SAGYOQ; Al-abbasi *et al.*, 2012 ▸). Compound HESLEX also involves a piperidine ring with a chair conformation linked by the C(=S)—N(H)—C(=O) bridge to a 3,4,5-tri­meth­oxy­benzene ring. It crystallizes with two independent mol­ecules in the asymmetric unit with slightly different conformations. For example, the mean plane of the piperidine rings are inclined to the benzene rings by 58.97 (11) and 64.11 (11)°, compared to 63.13 (3)° in the title compound. The central N—C(=S)—N(H)—C(=O) bridge is twisted in each compound, with an N2—C8—N1—C7 torsion angle of 74.8 (6)° in MPiCB, 63.0 (3)° in DUDYOS, 65.5 (3) and 79.9 (3)° in HESLEX, and finally −59.7 (2)° in SAGYOQ.

## Synthesis and crystallization   

Benzoyl chloride (0.01 mol) was added slowly to ammonium thio­cyanate (0.01 mol) in acetone and the mixture was stirred for 30 min at room temperature. A white precipitate of ammonium chloride was filtered off and the filtrate was cooled in an ice bath (278–283 K) for about 15 min. A cold solution (278–283 K) of piperidine (0.01 mol) in acetone was added to the benzoyl iso­thio­cyanate and the mixture was left for 3 h at room temperature. A yellowish precipitate was formed, filtered and washed with cold water to give pale-yellow crystals (yield 87%, m.p. 401-402 K).

The infrared spectrum of MPiCB shows the characteristic signals for ν(NH) 3300, ν(O—CH_3_) 2900, ν(C=O) 1609, ν(C—C_benzene_) 1460, ν(C—O_stretching_) 1327 and *v*(C=S) 1252 cm^−1^. The ^1^H NMR spectrum exhibits the H(N) group at 8.35 Hz, while the ^13^C NMR signal of the C=S and C=O groups appear at 174.66 and 163.19 Hz, respectively.

## Refinement   

Crystal data, data collection and structure refinement details are summarized in Table 2 ▸. The NH H atom was located in a difference-Fourier map and freely refined. The C-bound H atoms were included in calculated positions and refined in a riding-model approximation: C—H = 0.93–0.97 Å with *U*_iso_(H) = 1.2*U*_eq_(C).

## Supplementary Material

Crystal structure: contains datablock(s) I, Global. DOI: 10.1107/S2056989017013317/su4162sup1.cif

Structure factors: contains datablock(s) I. DOI: 10.1107/S2056989017013317/su4162Isup2.hkl

Supporting information file. DOI: 10.1107/S2056989017013317/su4162Isup3.cml

CCDC reference: 1575129

Additional supporting information:  crystallographic information; 3D view; checkCIF report

## Figures and Tables

**Table 1:** Hydrogen-bond geometry (Å, °) *Cg* is the centroid of the C1–C6 benzene ring.

*D*—H⋯*A*	*D*—H	H⋯*A*	*D*⋯*A*	*D*—H⋯*A*
N1—H1⋯O1^i^	0.84 (4)	2.12 (4)	2.897 (4)	154 (4)
C6—H6⋯O1^i^	0.93	2.40	3.294 (5)	160
C10—H10*A*⋯*Cg* ^ii^	0.97	2.89	3.851 (8)	170

Symmetry codes: (i) 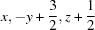; (ii) 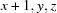.

**Table 2:** Experimental details

Crystal data	
Chemical formula	C_14_H_18_N_2_O_2_S
*M*_r_	278.36
Crystal system, space group	Monoclinic, *P*2_1_/*c*
Temperature (K)	296
*a*, *b*, *c* (Å)	8.2228 (9), 18.1289 (19), 9.945 (1)
β (°)	106.612 (3)
*V* (Å^3^)	1420.6 (3)
*Z*	4
Radiation type	Mo *K*α
μ (mm^−1^)	0.23
Crystal size (mm)	0.50 × 0.35 × 0.16

Data collection	
Diffractometer	Bruker SMART APEX CCD area-detector
Absorption correction	Multi-scan (*SADABS*; Bruker, 2007 ▸)
*T*_min_, *T*_max_	0.895, 0.965
No. of measured, independent and observed [*I* > 2σ(*I*)] reflections	38784, 2500, 1955
*R*_int_	0.049
(sin θ/λ)_max_ (Å^−1^)	0.595

Refinement	
*R*[*F* ^2^ > 2σ(*F* ^2^)], *wR*(*F* ^2^), *S*	0.081, 0.251, 1.03
No. of reflections	2500
No. of parameters	178
H-atom treatment	H atoms treated by a mixture of independent and constrained refinement
Δρ_max_, Δρ_min_ (e Å^−3^)	0.99, −0.52

Computer programs: *SMART* and *SAINT* (Bruker, 2007 ▸), *SHELXS97* (Sheldrick, 2008 ▸), *SHELXL97* (Sheldrick, 2015 ▸), *OLEX2* (Dolomanov *et al.*, 2009 ▸), *PLATON* (Spek, 2009 ▸) and *publCIF* (Westrip, 2010 ▸).

## supplementary crystallographic information

### Crystal data

**Table:**

C_14_H_18_N_2_O_2_S	*F*(000) = 592
*M**_r_* = 278.36	*D*_x_ = 1.301 Mg m^−^^3^
Monoclinic, *P*2_1_/*c*	Melting point = 402–401 K
Hall symbol: -P 2ybc	Mo *K*α radiation, λ = 0.71073 Å
*a* = 8.2228 (9) Å	Cell parameters from 9933 reflections
*b* = 18.1289 (19) Å	θ = 3.1–25.0°
*c* = 9.945 (1) Å	µ = 0.23 mm^−^^1^
β = 106.612 (3)°	*T* = 296 K
*V* = 1420.6 (3) Å^3^	Block, pale-yellow
*Z* = 4	0.50 × 0.35 × 0.16 mm

### Data collection

**Table:**

Bruker SMART APEX CCD area-detector diffractometer	2500 independent reflections
Radiation source: fine-focus sealed tube	1955 reflections with *I* > 2σ(*I*)
Graphite monochromator	*R*_int_ = 0.049
ω scan	θ_max_ = 25.0°, θ_min_ = 3.1°
Absorption correction: multi-scan (SADABS; Bruker, 2007)	*h* = −9→9
*T*_min_ = 0.895, *T*_max_ = 0.965	*k* = −21→21
38784 measured reflections	*l* = −11→11

### Refinement

**Table:**

Refinement on *F*^2^	Secondary atom site location: difference Fourier map
Least-squares matrix: full	Hydrogen site location: inferred from neighbouring sites
*R*[*F*^2^ > 2σ(*F*^2^)] = 0.081	H atoms treated by a mixture of independent and constrained refinement
*wR*(*F*^2^) = 0.251	*w* = 1/[σ^2^(*F*_o_^2^) + (0.1213*P*)^2^ + 2.9951*P*] where *P* = (*F*_o_^2^ + 2*F*_c_^2^)/3
*S* = 1.03	(Δ/σ)_max_ < 0.001
2500 reflections	Δρ_max_ = 0.99 e Å^−^^3^
178 parameters	Δρ_min_ = −0.52 e Å^−^^3^
0 restraints	Extinction correction: SHELXL97 (Sheldrick, 2008), Fc^*^=kFc[1+0.001xFc^2^λ^3^/sin(2θ)]^-1/4^
Primary atom site location: structure-invariant direct methods	Extinction coefficient: 0.012 (4)

### Special details

**Table:**

Geometry. All esds (except the esd in the dihedral angle between two l.s. planes) are estimated using the full covariance matrix. The cell esds are taken into account individually in the estimation of esds in distances, angles and torsion angles; correlations between esds in cell parameters are only used when they are defined by crystal symmetry. An approximate (isotropic) treatment of cell esds is used for estimating esds involving l.s. planes.
Refinement. Refinement of F^2^ against ALL reflections. The weighted R-factor wR and goodness of fit S are based on F^2^, conventional R-factors R are based on F, with F set to zero for negative F^2^. The threshold expression of F^2^ > 2sigma(F^2^) is used only for calculating R-factors(gt) etc. and is not relevant to the choice of reflections for refinement. R-factors based on F^2^ are statistically about twice as large as those based on F, and R- factors based on ALL data will be even larger.

### Fractional atomic coordinates and isotropic or equivalent isotropic displacement parameters (Å^2^)

**Table:**

	*x*	*y*	*z*	*U*_iso_*/*U*_eq_	
S1	0.10498 (18)	0.58727 (6)	1.00073 (14)	0.0680 (5)	
O1	0.0919 (5)	0.77247 (17)	0.7982 (3)	0.0700 (10)	
O2	−0.3058 (4)	1.02962 (17)	0.9922 (3)	0.0636 (9)	
N1	0.1299 (4)	0.73279 (18)	1.0176 (3)	0.0460 (8)	
N2	0.3615 (6)	0.6709 (2)	0.9800 (6)	0.0807 (15)	
C1	−0.0338 (5)	0.8445 (2)	0.9412 (4)	0.0416 (9)	
C2	−0.1170 (5)	0.8893 (3)	0.8297 (4)	0.0529 (11)	
H2	−0.1117	0.8781	0.7397	0.064*	
C3	−0.2068 (6)	0.9496 (3)	0.8504 (4)	0.0587 (11)	
H3	−0.2622	0.9788	0.7742	0.070*	
C4	−0.2165 (5)	0.9679 (2)	0.9832 (4)	0.0487 (10)	
C5	−0.1338 (5)	0.9242 (2)	1.0951 (4)	0.0496 (10)	
H5	−0.1389	0.9357	1.1850	0.059*	
C6	−0.0434 (5)	0.8633 (2)	1.0739 (4)	0.0482 (10)	
H6	0.0123	0.8342	1.1502	0.058*	
C7	0.0655 (5)	0.7812 (2)	0.9123 (4)	0.0460 (10)	
C8	0.2076 (6)	0.6650 (2)	0.9959 (4)	0.0502 (10)	
C9	0.4655 (9)	0.7393 (3)	0.9999 (9)	0.102 (2)	
H9A	0.3928	0.7814	0.9997	0.122*	
H9B	0.5499	0.7374	1.0907	0.122*	
C10	0.5469 (9)	0.7491 (4)	0.8969 (9)	0.105 (2)	
H10A	0.6200	0.7920	0.9201	0.126*	
H10B	0.4624	0.7588	0.8082	0.126*	
C11	0.6528 (7)	0.6829 (4)	0.8795 (8)	0.0910 (18)	
H11A	0.6892	0.6889	0.7956	0.109*	
H11B	0.7533	0.6796	0.9593	0.109*	
C12	0.5502 (10)	0.6128 (4)	0.8685 (10)	0.119 (3)	
H12A	0.4694	0.6110	0.7759	0.143*	
H12B	0.6262	0.5711	0.8762	0.143*	
C13	0.4638 (11)	0.6046 (3)	0.9654 (10)	0.119 (3)	
H13A	0.5442	0.5940	1.0557	0.143*	
H13B	0.3885	0.5625	0.9396	0.143*	
C14	−0.3187 (7)	1.0502 (3)	1.1266 (6)	0.0678 (13)	
H14A	−0.3689	1.0106	1.1649	0.102*	
H14B	−0.3884	1.0934	1.1179	0.102*	
H14C	−0.2076	1.0605	1.1877	0.102*	
H1	0.085 (5)	0.732 (2)	1.084 (4)	0.043 (11)*	

### Atomic displacement parameters (Å^2^)

**Table:**

	*U*^11^	*U*^22^	*U*^33^	*U*^12^	*U*^13^	*U*^23^
S1	0.0856 (10)	0.0478 (7)	0.0731 (9)	−0.0160 (6)	0.0266 (7)	0.0031 (5)
O1	0.124 (3)	0.0583 (19)	0.0390 (16)	−0.0046 (18)	0.0422 (17)	−0.0021 (13)
O2	0.0632 (19)	0.0547 (18)	0.070 (2)	0.0078 (15)	0.0152 (15)	0.0078 (15)
N1	0.064 (2)	0.0447 (19)	0.0374 (17)	−0.0028 (15)	0.0268 (16)	−0.0016 (14)
N2	0.094 (3)	0.042 (2)	0.133 (4)	−0.009 (2)	0.075 (3)	−0.019 (2)
C1	0.049 (2)	0.042 (2)	0.0356 (19)	−0.0123 (16)	0.0146 (16)	0.0004 (15)
C2	0.062 (2)	0.063 (3)	0.032 (2)	−0.008 (2)	0.0096 (17)	0.0012 (18)
C3	0.060 (3)	0.065 (3)	0.044 (2)	0.001 (2)	0.0033 (19)	0.012 (2)
C4	0.043 (2)	0.046 (2)	0.055 (2)	−0.0077 (17)	0.0115 (17)	0.0005 (18)
C5	0.062 (2)	0.050 (2)	0.040 (2)	−0.0004 (19)	0.0181 (18)	−0.0006 (17)
C6	0.064 (2)	0.048 (2)	0.033 (2)	0.0011 (19)	0.0133 (17)	0.0059 (16)
C7	0.064 (2)	0.047 (2)	0.0309 (19)	−0.0177 (18)	0.0199 (17)	−0.0060 (16)
C8	0.070 (3)	0.044 (2)	0.042 (2)	−0.0095 (19)	0.0253 (19)	−0.0063 (16)
C9	0.103 (5)	0.054 (3)	0.175 (7)	−0.021 (3)	0.084 (5)	−0.028 (4)
C10	0.084 (4)	0.079 (4)	0.174 (7)	−0.015 (3)	0.075 (5)	−0.011 (4)
C11	0.064 (3)	0.095 (4)	0.124 (5)	0.012 (3)	0.041 (3)	−0.005 (4)
C12	0.094 (5)	0.088 (5)	0.189 (8)	0.018 (4)	0.064 (5)	−0.036 (5)
C13	0.142 (6)	0.055 (3)	0.195 (9)	0.010 (4)	0.105 (6)	−0.010 (4)
C14	0.069 (3)	0.053 (3)	0.087 (4)	0.004 (2)	0.031 (3)	−0.006 (2)

### Geometric parameters (Å, º)

**Table:**

S1—C8	1.651 (4)	C5—H5	0.9300
O1—C7	1.226 (4)	C6—H6	0.9300
O2—C4	1.356 (5)	C9—C10	1.385 (9)
O2—C14	1.421 (6)	C9—H9A	0.9700
N1—C7	1.352 (5)	C9—H9B	0.9700
N1—C8	1.430 (5)	C10—C11	1.521 (8)
N1—H1	0.84 (4)	C10—H10A	0.9700
N2—C8	1.323 (6)	C10—H10B	0.9700
N2—C9	1.487 (6)	C11—C12	1.511 (9)
N2—C13	1.497 (7)	C11—H11A	0.9700
C1—C2	1.386 (6)	C11—H11B	0.9700
C1—C6	1.387 (5)	C12—C13	1.358 (10)
C1—C7	1.484 (6)	C12—H12A	0.9700
C2—C3	1.367 (6)	C12—H12B	0.9700
C2—H2	0.9300	C13—H13A	0.9700
C3—C4	1.386 (6)	C13—H13B	0.9700
C3—H3	0.9300	C14—H14A	0.9600
C4—C5	1.377 (6)	C14—H14B	0.9600
C5—C6	1.381 (6)	C14—H14C	0.9600
			
C4—O2—C14	117.8 (3)	C10—C9—H9B	109.0
C7—N1—C8	122.2 (3)	N2—C9—H9B	109.0
C7—N1—H1	117 (3)	H9A—C9—H9B	107.8
C8—N1—H1	115 (3)	C9—C10—C11	113.3 (6)
C8—N2—C9	125.8 (4)	C9—C10—H10A	108.9
C8—N2—C13	122.0 (4)	C11—C10—H10A	108.9
C9—N2—C13	111.3 (5)	C9—C10—H10B	108.9
C2—C1—C6	117.9 (4)	C11—C10—H10B	108.9
C2—C1—C7	118.1 (3)	H10A—C10—H10B	107.7
C6—C1—C7	123.9 (3)	C12—C11—C10	110.2 (5)
C3—C2—C1	120.8 (4)	C12—C11—H11A	109.6
C3—C2—H2	119.6	C10—C11—H11A	109.6
C1—C2—H2	119.6	C12—C11—H11B	109.6
C2—C3—C4	121.0 (4)	C10—C11—H11B	109.6
C2—C3—H3	119.5	H11A—C11—H11B	108.1
C4—C3—H3	119.5	C13—C12—C11	115.8 (6)
O2—C4—C5	124.9 (4)	C13—C12—H12A	108.3
O2—C4—C3	116.1 (4)	C11—C12—H12A	108.3
C5—C4—C3	119.0 (4)	C13—C12—H12B	108.3
C4—C5—C6	119.9 (4)	C11—C12—H12B	108.3
C4—C5—H5	120.0	H12A—C12—H12B	107.4
C6—C5—H5	120.0	C12—C13—N2	113.8 (6)
C5—C6—C1	121.4 (4)	C12—C13—H13A	108.8
C5—C6—H6	119.3	N2—C13—H13A	108.8
C1—C6—H6	119.3	C12—C13—H13B	108.8
O1—C7—N1	120.0 (4)	N2—C13—H13B	108.8
O1—C7—C1	122.1 (4)	H13A—C13—H13B	107.7
N1—C7—C1	117.9 (3)	O2—C14—H14A	109.5
N2—C8—N1	115.7 (3)	O2—C14—H14B	109.5
N2—C8—S1	125.9 (3)	H14A—C14—H14B	109.5
N1—C8—S1	118.4 (3)	O2—C14—H14C	109.5
C10—C9—N2	112.9 (6)	H14A—C14—H14C	109.5
C10—C9—H9A	109.0	H14B—C14—H14C	109.5
N2—C9—H9A	109.0		
			
C6—C1—C2—C3	−0.5 (6)	C2—C1—C7—N1	−172.4 (4)
C7—C1—C2—C3	−178.3 (4)	C6—C1—C7—N1	10.0 (6)
C1—C2—C3—C4	0.3 (7)	C9—N2—C8—N1	7.8 (8)
C14—O2—C4—C5	−1.5 (6)	C13—N2—C8—N1	176.1 (6)
C14—O2—C4—C3	180.0 (4)	C9—N2—C8—S1	−168.8 (5)
C2—C3—C4—O2	178.7 (4)	C13—N2—C8—S1	−0.6 (9)
C2—C3—C4—C5	0.0 (6)	C7—N1—C8—N2	74.8 (6)
O2—C4—C5—C6	−178.6 (4)	C7—N1—C8—S1	−108.2 (4)
C3—C4—C5—C6	−0.1 (6)	C8—N2—C9—C10	−137.5 (7)
C4—C5—C6—C1	−0.2 (6)	C13—N2—C9—C10	53.2 (9)
C2—C1—C6—C5	0.5 (6)	N2—C9—C10—C11	−53.7 (9)
C7—C1—C6—C5	178.1 (4)	C9—C10—C11—C12	48.7 (9)
C8—N1—C7—O1	−9.5 (6)	C10—C11—C12—C13	−47.0 (10)
C8—N1—C7—C1	171.5 (3)	C11—C12—C13—N2	49.2 (11)
C2—C1—C7—O1	8.6 (6)	C8—N2—C13—C12	139.6 (7)
C6—C1—C7—O1	−169.0 (4)	C9—N2—C13—C12	−50.6 (10)

### Hydrogen-bond geometry (Å, º)

Cg is the centroid of the C1–C6 benzene ring.

**Table:**

*D*—H···*A*	*D*—H	H···*A*	*D*···*A*	*D*—H···*A*
N1—H1···O1^i^	0.84 (4)	2.12 (4)	2.897 (4)	154 (4)
C6—H6···O1^i^	0.93	2.40	3.294 (5)	160
C10—H10*A*···*Cg*^ii^	0.97	2.89	3.851 (8)	170

Symmetry codes: (i) *x*, −*y*+3/2, *z*+1/2; (ii) *x*+1, *y*, *z*.
